# The Use of Interactions Between Microorganisms in Strawberry Cultivation (*Fragaria x ananassa* Duch.)

**DOI:** 10.3389/fpls.2021.780099

**Published:** 2021-11-29

**Authors:** Magdalena Drobek, Justyna Cybulska, Anna Gałązka, Beata Feledyn-Szewczyk, Anna Marzec-Grządziel, Lidia Sas-Paszt, Agata Gryta, Paweł Trzciński, Artur Zdunek, Magdalena Frąc

**Affiliations:** ^1^Institute of Agrophysics, Polish Academy of Sciences, Lublin, Poland; ^2^The Institute of Soil Science and Plant Cultivation (IUNG)–State Research Institute, Puławy, Poland; ^3^National Institute of Horticultural Research, Skierniewice, Poland

**Keywords:** antagonistic microorganisms, pectin, strawberry, organic fruit, biostimulants

## Abstract

As the market indicates a growing interest in organically grown fruit, there is a need for biostimulants to counter the adverse effects of pathogenic fungi and fungal-like-pathogens. Four microbial pathogens (*Botrytis cinerea*, *Verticillium* sp., *Phytophthora* sp., and *Colletotrichum* sp.) which are the most often causes of strawberry diseases were selected. Five kinds of biostimulants (C1, C2, C3, C4, and C5) containing bacterial consortia were developed to combat the pathogens. The antagonistic effect of selected microorganisms against strawberry pathogens was observed. The effectiveness of various beneficial bacteria in combating fungal pathogens of cv. Honeoye strawberries was compared and the impact of their activity on fruit quality was assessed. The most significant effect on the strawberry firmness was found for the C2 consortium, which provided the strawberries infected with the pathogens group (MIX: *B. cinerea*, *Verticillium* sp., *Phytophthora* sp., and *Colletotrichum* sp.) with a 140% increase in maximum load in a puncture test compared to the positive control (C0). Strawberries contaminated with *Phytophthora* sp. after the application of Consortium C4 (C4) showed the largest increase (127%) in soluble solid content (SSC) when compared to the C0. Fruit contaminated with *Colletotrichum* sp. and *B. cinerea* after the application of C2 and Consortium 5 (C5), respectively, had the highest levels of anthocyanins and total phenolic content, when compared to C0. The largest increase, which reached as high as 25%, in D-galacturonic acid content was observed for the group of pathogens after Consortium 1 (C1) application. The extraction of strawberry pectin allowed for the study of the rheological properties of pectin solutions; on this basis, strawberry pectin from the control (NC) was distinguished as it showed the highest viscosity (0.137–0.415 Pas). Taking into account the individual effects of bacteria on strawberry pathogenic fungi and fungal-like-pathogens, it is possible to reduce the adverse effects of fungal disease and to improve the properties of strawberries by selecting the appropriate bacterial consortium. Interactions between microorganisms are often complex and not fully understood, which suggests the need for further research in this direction.

## Introduction

The strawberry is one of the most frequently preferred fruit by consumers due to availability year around and suitable for both organic and conventional production (Balci, 2021; Juric et al., 2021). Excessive pesticide use in conventional crops may lead to their accumulation in the fruit. For this reason, alternative control measures, which include the biopesticides or antagonistic microorganisms (Jensen et al., 2013). The organic farming system differs from the conventional one in prohibition of synthetic plant protection products (pesticides) and mineral fertilizers. The chemical plant protection products are replaced with organic fertilizers, which contain, among others, manure, compost, green manure, beneficial microorganisms (Ponder and Hallmann, 2019). In the context of organic fertilizers, the concept of biostimulants appears. Biostimulants are defined by the European Biostimulants Industry Council as “substance (s) and/or microorganisms whose function, when applied to plants or the rhizosphere, is to stimulate natural processes to increase/benefit nutrient uptake, nutrient efficiency, tolerance to abiotic stress, and quality of the crops” (European Biostimulants Industry Council [EBIC], 2021). It has been proven that biostimulants increase plant yield and improve fruit quality. Additionally, consumers are more interested in ecological products each year, bearing in mind the safety of food and the environment (Kovačević et al., 2015).

The microbiota present on healthy strawberries is complex and includes potential plant pathogens, human pathogens, mycotoxin-producing moulds and plant disease biocontrol agents (Jensen et al., 2013). Some of these microorganisms are antagonistic to the others. Notable bacterial antagonism against pathogenic fungi occurs with considerable frequency and is the result, among other causes, of the production of antibiotics and biosurfactants, as well as competition and parasitism (Berg et al., 2000). The mechanisms of action differ depending on the bacterial strain. The most common strawberry pathogens include fungi from the genera *Botrytis*, *Verticillium*, *Phytophthora*, *Colletotrichum*, *Penicillium*, *Alternaria*, *Cladosporium*, *Rhizopus*, *Aureobasidium*, and *Cryptococcus* (Tournas and Katsoudas, 2005). Bacterial populations on strawberry plants are dominated by *Pseudomonas* spp., *Stenotrophomonas* spp., *Bacillus* spp., and *Arthrobacter* spp. (Krimm et al., 2005).

One of the main strawberry postharvest diseases is grey mould which is caused by *Botrytis cinerea*. A grey coating appears on the leaves and fruit causing the plants to die off and the fruit to dry and rot (Jin et al., 2017). Research results suggest that the following microorganisms are active in combating grey rot: *Clonostachys rosea* (Cota et al., 2009), *Rhodotorula glutinis* (Srivastava et al., 2011), *Paenibacillus polymyxa* (Santiago et al., 2016), *Bacillus* sp. (Cruz et al., 2018), *Streptomyces* sp. (Kim et al., 2015), and *Pseudomonas* sp. (Haggag and Abo El Soud, 2012). The withering of strawberry plants is mainly caused by *Verticillium dahliae*, which attacks the plant’s vascular system and blocks the transport of water and nutrients (Sowik et al., 2001). Research indicates that *P. polymyxa* (Zhang et al., 2018), *Bacillus* sp. (Milijasevic-Marcic et al., 2018), and *Streptomyces* sp. (Olanrewaju and Babalola, 2019) are disease-inhibiting bacteria. Fungus-like pathogens of the genus *Phytophthora* including *Phytophthora megasperma*, *Phytophthora cryptogea*, *Phytophthora capsici*, *Phytophthora Citricola*, and *Phytophthora cactorum* can cause diseases of the root, crown and strawberry fruit. Infected plants suddenly lose healthy shoots. A relatively rare disease is red stele in strawberries (*Phytophthora fragariae*). Infected plants become stunted and the leaves turn yellow or red (Wilcox et al., 1993). *Streptomyces griseus* has shown promising results as an antifungal agent against *P. capsici*, as it produces numerous antibiotics that inhibit mycelium growth of *P. capsici*, *Pythium* spp., *Phytophthora* spp., *Rhizoctonia solani*, *Alternaria brassicicola*, and *Botrytis* sp. (Nguyen et al., 2015). The fungi belonging to the species of *Colletotrichum asianum*, *Colletotrichum fructicola*, *Colletotrichum tropicale*, *Colletotrichum dianesei*, *Colletotrichum karstii* (Lima et al., 2013), *Colletotrichum gloeosporioides* (Tang et al., 2019), and *Colletotrichum acutatum* (Moreira et al., 2014) cause anthracnose of fruit, which in the case of strawberries is manifested by the appearance of dry spots on immature fruit, and the browning and drying of flowers and shoots (Lima et al., 2013). It has been shown that the fungi *Aureobasidium pullulans*, *Diaporthe* sp., and *Nigrospora oryzae* inhibits the development of *C. acutatum* (Landum et al., 2016). Useful fungi and bacteria limit the growth and development of pathogens by activating antagonistic mechanisms such as parasitism, antibiotics, and competition (Poveda et al., 2020).

We investigated the impact of various biostimulants containing fungi and bacteria on the quality characteristics of strawberry fruit infected with microbial pathogens. The experiment included four fungal pathogens responsible for the most common strawberry diseases. Bacterial consortia with potentially antagonistic effects were applied to the infected plants infected with *B. cinerea, Verticillium* sp., *Phytophthora* sp., and *Colletotrichum* sp. The results allow for a preliminary selection of microorganisms that can be used to combat strawberry fungal diseases.

## Materials and Methods

### Crop Conditions

**FIGURE 1:**
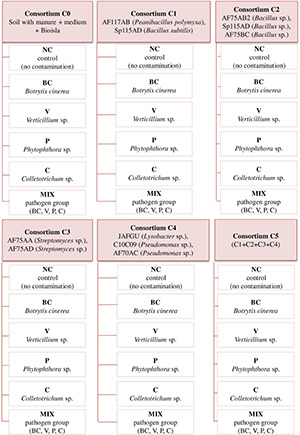
Experimental design outlining 36 variants of the experiment.

The experiment was set up in triplicate for each of the 36 variants (Figure 1) and the research material consisted of strawberry cv. Honeoye. Each strawberry plant was grown in a separate pot without undercutting or root curling in a greenhouse at 23°C. Each pot contained 4 kg of soil collected from an organic experimental field of the Institute of Soil Science and Plant Cultivation - State Research Institute (Puławy, Poland) located in Grabów (Poland) and it was subjected to chemical and microbiological analysis. The soil was characterized as Cambisol with a loamy sand texture. Clover was used as a pre-crop. The soil composition was as follows: humus 1.9%, P_2_O_5_ 10.1 mg 100 g^–1^ soil, K_2_O 5.0 mg 100 g^–1^ soil, Mg 10.6 mg 100 g^–1^ soil, N-NO_3_ 8.92 mg kg^–1^ DM of soil, N-NH_4_ 2.29 mg kg^–1^ DM of soil, pH 5.9. Keramzite was placed at the bottom of each pot in a volume of 0.5 l. The soil was fertilized in autumn with manure (pH = 8.6, nutrient content: N 5.06 mg kg^–1^, P_2_O_5_ 2.47 mg kg^–1^, K_2_O 6.90 mg kg^–1^, CaO 3.04 mg kg^–1^, MgO 2.38 mg kg^–1^, S 0.69 mg kg^–1^) and potassium salt (LUVENA, 100 kg ha^–1^). Bioilsa^®^ (NaturalCrop, Poland) fertilizer was applied in a quantity corresponding to a dose of 150 kg ha^–1^.

Plants were treated with five consortia (C1, C2, C3, C4, and C5) and a control (C0) was prepared, in which no consortia were applied (Figure 1). The consortia consisted of the following bacterial strains: AF117AB (*P. polymyxa*), Sp115AD (*Bacillus subtilis*), AF75AB2, Sp115AD, AF75BC (*Bacillus* sp.), AF75AA, AF75AD (*Streptomyces* sp.), JAFGU (*Lysobacter* sp.), and AF70AC (*Pseudomonas* sp.). The bacterial strains used in the experiment originated from the SYMBIOBANK collection of the Research Institute of Horticulture in Skierniewice and were selected based on a previous study as microorganisms with potentially beneficial properties in terms of plant biostimulation and protection. The bacterial consortia, with a population size of 10^8^ cfu (colony-forming units) in 1 ml, were applied to the soil in a volume of 10 ml per pot directly to the roots at planting time and twice after planting at intervals of 3–4 weeks. The objects called “Consortium C0,” in which bacteria were not introduced, were the control for the ones containing bacterial consortia with their potential for plant biostimulation and protection, while NC objects constituted controls without pathogen contamination.

The next step was the infection of plants with five phytopathogenic fungi and fungus-like pathogens (BC, V, P, C, and MIX) and the submission of the control (NC) in which the plants were not treated with pathogens (Figure 1). Two days after plant inoculation with bacterial consortia, the plants were infected with the following pathogens: G277/18 (*Botrytis cinerea*), G296/18 (*Verticillium* sp.), G408/18 (*Phytophthora* sp.), G171/18 (*Colletotrichum* sp.). Strains of *B. cinerea*, *Phytophthora* sp., and *Verticillium* sp. were isolated from infected strawberry roots (Oszust et al., 2020), while *Colletotrichum* sp. was isolated from infected strawberry fruit (Malarczyk et al., 2020) at the Institute of Agrophysics of the Polish Academy of Sciences (Lublin, Poland). In the case of *B. cinerea* and *Colletotrichum* sp. the strawberry flowers were infected with 10 ml of pathogen suspension using an inoculum adjusted to 1,000 conidia in 1 ml. In contrast, *Phytophthora* sp. and *Verticillium* sp. were added to the soil as a pathogen inoculum suspension of 10^5^ cfu ml^–1^ (10 ml was applied) and 10^4^ cfu g^–1^ of soil, respectively.

At the harvest maturity stage, the strawberries (developed and grown, and the sepals easily detach from the stalk) were collected on March 23, 2019.

### Firmness

Firmness is one of main parameter of texture which determine overall quality evaluation by consumers (Harker et al., 2003). For soft fruit puncture test is the most common method used for determination of firmness (Døving and Måge, 2002). Fresh fruit firmness (n) was determined by a puncture test by using a universal test machine (Lloyd LRX, Lloyd Instruments). The strawberry was placed on the platform of the device and the aluminium probe (3 mm diameter) was lowered with a constant plunger speed (20 mm min^–1^) to a constant depth (6 mm). The maximum force required to penetrate the strawberry to a set depth was a measure of tissue firmness (Fraeye et al., 2009). The test was carried out on twenty strawberries for each replicate of the variant. Average fruit firmness was expressed in terms of *N*.

### Shape

The shape of the fruit is a hormone-regulated trait and many factors can alter the final shape including environmental conditions (Rey-Serra et al., 2021). The shape was determined in accordance with the method proposed by Ishikawa et al. (2018). The length (the longer dimension was measured from the fruit’s tip to the base) and width (the shorter dimension of the fruit, was measured as a transverse measurement at the thickest point) of the fruit were determined. The quotient of length and width was calculated. Fruits are classified as flattened (quotient < 1), round (quotient = 1), or oblong (quotient > 1). The test was carried out on fifteen strawberries for each replicate of the variant.

### Fresh Weight of Fruit

Fresh weight is the weight of the fruit on the day it is harvested. It is determined by a weight method to initially determine the size of the fruit (Reyes-Yanes et al., 2020). The purpose of determining the fresh weight is to obtain information on the effectiveness of the cultivation methods. Comparison the fresh weight, dry weight and shape provides information on the hydration level of the fruit and the tissue density (Miller et al., 2000). To determine the fresh weight of fruit (FWF), each fruit was weighed to three decimal places. The results were summed and averaged. The average fresh fruit weight was expressed in g.

### Dry Weight of Fruit

The dry matter allows the determination of the product residue after the removal of water from it (Ozdemir and Topuz, 2004). The drying oven method is a validated standardized method that is readily available (International Organization for Standardization, 2000). Dry weight (DW) was determined according to the procedure described in standard PN ISO 1026:2000 (International Organization for Standardization, 2000). In Brief, 0.5 g of strawberries were taken from each variant in triplicate and weighed to the nearest 0.001 g. The strawberries were then placed in an oven at 105°C, after 24 h the fruit was weighed, placed in the oven for 2 h and weighed again. The procedure was repeated until no further weight loss was noted. The average dry weight of one fruit was expressed as a percentage of fresh weight.

### Soluble Sugar Content

The soluble solid content (SSC) is a measure of the total soluble solid, which includes sugars, organic acids, amino acids, and more, and it is associated with consumer preference for fruit (Crisosto et al., 2003). The refractometric method is used because of the optical properties of sugars and sugar alcohols which are majority of soluble solids of most fruit (Magwaza and Opara, 2015). SSC was determined using a refractometer (PAL-BX/RI, ATAGO, Japan) and expressed in percentage terms. A minimum of five fresh strawberries were picked and juiced. One drop of juice was applied to the refractometer glass and the measurement was performed; the soluble sugar content was expressed in percentage terms. The measurement was performed in triplicate for each variant of the experiment.

### Total Phenolic Content

Due to the strong antioxidant activity of most phenolic compounds in fruits, the total polyphenol content is determined for the evaluation of health-promoting properties. The Folin-Ciocalteu method has been used for many years and is still relevant today due to its high accuracy (Carmona-Hernandez et al., 2021). Phenolic compounds (PC) were determined according to the procedure proposed by Folin and Ciocalteu (1927) and Nunes et al. (2001), with some modifications. From each variant of the experiment, five fruit were taken in triplicate and then juiced. Then, 0.5 ml of the clear juice was diluted by adding 9.5 ml of deionized water. The resulting solution was spiked with 5 ml of Folin-Ciocalteu reagent (1:9 dilution) and 4 ml of sodium carbonate (0.075 g ml^–1^). The mixture was left to stand for 30 min at 30°C, and then transferred to an ice bath (0°C) for 30 min. The absorbance of the solutions was measured at 760 nm. The content of phenolic compounds was determined using gallic acid solution as a standard, and the phenol concentration was expressed in mg 100 g^–1^ FW.

### Anthocyanins Content

Anthocyanin is natural dye responsible for the color of the fruit and belongs to antioxidants which demonstrate health beneficial properties. The spectrophotometric method based on the reversible discoloration of anthocyanin depending on the pH is commonly used to measure the anthocyanin content (Johnson et al., 2020). The anthocyanins content (AC) was determined according to a previous procedure described by Nunes et al. (2001) and consisted of measuring the content of pelargonidin-3-glucoside [the main pigment in strawberries (Nunes et al., 2001)]. For each variant, 2 g of strawberry pulp was collected in triplicate. Subsequently, 18 ml of 0.5% (v/v) HCl in methanol was added to the pulp and the mixture was incubated in a refrigerator (4°C) for 1 h to extract the pigment. After incubation, the mixture was centrifuged and the supernatant was used to determine the anthocyanin content through the use of spectrophotometry at 520 nm. The anthocyanin content was calculated using the following formula: *A*_520_^∗^ dilution factor ^∗^ [molecular weight of pelargonidin-3-glucoside (PGN)/molar extinction coefficient (MEC)], where PGN = 433.2, MEC = 2.908 ^∗^ 10^4^. The result was expressed in terms of an average of three repetitions in terms of mg 100 g^–1^ FW.

### Pectin Extraction

Pectin are one of the basic components of the cell wall. It has been shown that oxalate-soluble pectin play a significant role in shaping the texture of the fruit, which directly affects, among others, resistance to mechanical damage (Makarova and Shakhmatov, 2020). Strawberry pectin analysis was carried out according to the procedure proposed by Koubala et al. (2008) and Min et al. (2011) with some modifications. 1 g of pulp was taken in triplicate from each experimental variant. 35 ml of 0.25% ammonium oxalate (pH = 4.6) was added to the pulp and the mixture was transferred to a water bath (85°C) for 1 h. After incubation, the mixture was centrifuged (20,000 × *g*) for 10 min and the supernatant was collected. Three volumes of 96% ethanol were added to the supernatant and the solution was incubated in a refrigerator (4°C) for 24 h. The next step was to centrifuge (20,000 × *g*, 10 min) the solution and discard the supernatant. The remaining residue contained pectin, it was washed twice with 96% ethanol, dried and used to determine the D-galacturonic acid content.

### Galacturonic Acid Content

Content of galacturonic acid as the main component of pectin determines suitability of fruit for processing (gelling, production of ascorbic acid) (Ben Abdallah et al., 2018). The content of D-galacturonic acid (GalA) in strawberries was determined using a continuous flow analyser (CFA), SanPlus (Skalar, Netherlands) according to the procedure recommended by the manufacturer. The applied automatic colorimetric method of measurement of GalA content is based on a color reaction, where the color intensity is measured at a wavelength of 520 nm (Ognyanov et al., 2021). 10 mg of dried pectin was dissolved in 12 ml of deionised water. The resulting solution was diluted 10 times and the D-galacturonic acid content was determined. The result is expressed in g kg^–1^ DW.

### Rheological Properties

The rheological properties, which demonstrate the relationship between the structural, mechanical and sensory properties, are among the basic parameters of pectin (Xie et al., 2021). The rheological properties were measured using a Discovery HR-1 hybrid rheometer (TA Instruments, United States) with a cone-plate sensor. In brief, 1% aqueous solutions of strawberry pectin extracted with ammonium oxalate were prepared according to a procedure described elsewhere (Koubala et al., 2008; Min et al., 2011). For each variant of the experiment, pectin solutions were prepared in triplicate and each measurement was carried out at a temperature of 20 ± 0.5°C. Viscosity was measured at a constant shear rate of 10 s^–1^ (three repetitions), and the flow curves were determined based on measurements at variable shear rates of 10 and 600 s^–1^. Flow curves were described using the Ostwald–de Waele model as represented by the following equation:


$$\sigma =k\gamma^{\text{n}}$$

where σ is shear stress (Pa), *k* is consistency index (Pas^n^), γ is shear rate (s^−1^) and n is flow behaviour index.

### Antagonistic Properties of Consortia Against Fungal Pathogens

Antagonistic properties of consortia against fungal pathogens are determined in order to test the effectiveness of the tested bacterial consortia in combating fungal and fungal-like pathogens in the strawberry (Win et al., 2021). *In vitro* confrontations of four separate bacterial consortia and their mixtures (C5) against four selected phytopathogenic fungi and fungal-like-pathogens were set up using the plate culture method (Petri dish diameter 90 mm) with potato dextrose agar (PDA). Paper discs (5 mm diameter) were surface sterilized with UV light, dipped in 30 μl of bacterial consortia suspension (10^8^ cfu ml^–1^) and placed in duplicates on Petri dishes inoculated with fungal pathogens. The pathogen inoculation included 150 μl of inoculum of a 7-day old culture of *B. cinerea* G277/18, *Phytophthora* sp. G408/18, *Verticillium* sp. G296/18, and *Colletotrichum* sp. G171/18 suspended with 10 ml of sterile water. The concentration of *B. cinerea* and *Colletotrichum* sp. was 10^3^ jtk ml^–1^, *P. cactorum* 10^5^ jtk ml^–1^, and *Verticillium* sp. 10^4^ jtk g^–1^. The pathogen-inoculated plates with discs soaked in bacterial consortia were incubated at 26°C in the dark. The observations were recorded 4 days after inoculation by measuring the fungal growth inhibition zone around the paper discs containing bacterial consortia.

### Characteristics of Soil Used for Strawberry Cultivation

Characteristics of soil used for strawberry cultivation is a source of information about the processes during the growth and development of plants, which can have a direct impact on the quality of the fruit. Methods determining the content of proteins, enzymes, carbon and nitrogen in soil provide information on the level of soil degradation and allow the assessment of the importance of the biological consortia used (Wang et al., 2017). Soil samples were taken by complete randomization from a depth of 0–15 cm. The soil was dried at 23°C and sieved through a sieve with a mesh diameter of 2 mm. Total glomalin content (TG), easily extractable fractions of glomalin (EEG) (Wrigth and Upadhyaya, 1998), soil enzyme activity (dehydrogenase DHA) (Klein et al., 1964), acidic phosphatase (ACP), alkaline phosphatase (ALP) (Tabatabai and Bremner, 1969), as well as the carbon and nitrogen content in the biomass were determined in the samples.

### Principal Component Analysis

Principal component analysis (PCA) is used to discover regularities between variables. It consists in determining the components which are a linear combination of the examined variables. PCA enables the identification of those initial variables that have a significant impact on the appearance of individual principal components (Yagmur and Gunes, 2021). In order to assess the relationship between the studied parameters, PCA analysis was performed using STATISTICA software (Statistica v.12, StatSoft Inc., United States).

### Statistical Analysis

The obtained results were analysed with STATISTICA software (Statistica v.12, StatSoft Inc., United States) using a two-way analysis of variance (ANOVA) followed by the Tukey HSD test or Tuckey for different N, F-Welch and RIR Tuckey test for unequal numbers or ANOVA Kruskal–Wallis test; significant differences were determined at *P* < 0.05.

## Results and Discussion

Strawberries are in high demand because of their taste and health-promoting properties resulting from the presence of antioxidants and vitamins. The development of organic farming and the promotion of a healthy lifestyle has significantly increased the demand for organically grown fruit. The recent guidelines of the European Green Deal and Biodiversity Strategy have led to the use of sustainable practices, such as organic farming, and to a reduction in the use of chemical pesticides (European Commission, 2019). These measures have led to fruit producers ensuring the high quality of their strawberries by using natural plant protection preparations. For this purpose, plant extracts, microbial consortia that antagonize strawberry pathogens (Ornelas-Paz et al., 2013) and plant protective hormones (Poveda, 2020) are both desirable and beneficial in terms of biocontrol. For economic reasons, it is important that ecological preparations not only increase the quality of the fruit, but also guarantee a plentiful harvest, reasonable production costs and that the market price of the fruit should be taken into consideration. Therefore, bacterial strains with potentially antagonistic properties against main fungal and fungal-like pathogens were selected for this study.

### Strawberry Quality

**FIGURE 2:**
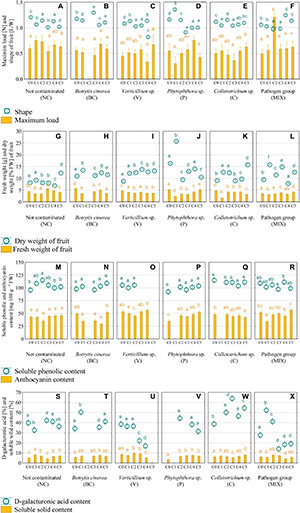
Firmness of strawberries and shape **(A–F)**, fresh and dry weight **(G–L)**, soluble phenolic and anthocyanin content **(M–R)**, and D-galacturonic acid and soluble solid content **(S–X)** in strawberry fruit. C0, control (not contaminated); C1, Consortium 1; C2, Consortium 2; C3, Consortium 3; C4, Consortium 4; C5, Consortium 5. A statistical analysis was performed for each pathogen group of plants separately. The data are means ± SD (*n* > 3). Different letters (a–f) indicate the differences between consortia (*P* < 0.05) as determined by the following statistical test: ANOVA Tuckey test for different N for maximum load **(C–E)** and shape **(A–F)**; F-Welch test and RIR Tuckey test for unequal numbers for **(A,B,F)**, ANOVA and Tuckey test HSD for fresh weight **(G,H,L)**, dry weight **(L)**, anthocyanin **(N–Q)**, soluble phenolic **(N–Q)**, D-galacturonic acid **(W)**, soluble solid content **(W,X)**; F-Welch and *post hoc* for fresh weight **(I–K)**, dry weight **(J,K)**, anthocyanin **(M,R)**, soluble phenolic **(M,R)**, D-galacturonic acid **(S–U,X)**, soluble solid content **(S–U)**; ANOVA Kruskal–Wallis test for dry weight **(G–I)**, D-galacturonic acid **(V)**, soluble solid content **(V)**.

The shape of strawberries has a significant influence over visual quality and consumer interest. Based on previous findings, auxin derived from achene is responsible for cell division and expansion (Zahedipour-Sheshglani and Asghari, 2020), this changes the shape of the strawberry. Consumers prefer round strawberries (Zahedipour-Sheshglani and Asghari, 2020). The change in shape from oblong (in C0) to round after the use of the appropriate consortium was noted in strawberry P (C4 and C5) and (Figure 2D), which makes them the most attractive to the consumer. Strawberry V (C4), P (C3), and MIX (C2) were distinguished by their flattened shape (Figures 2C,D,F). The remaining fruits were characterized as oblong.

An important parameter for assessing the quality of strawberries is firmness. Ensuring the proper firmness of the fruit allows for longer storage periods and facilitates transport over greater distances (Liu et al., 2019). The highest increase in firmness was observed for MIX-infected strawberries treated with C2, the maximum load in this case was on average 2.4 times higher than in the control (C0) (Figure 2F). A statistically significant increase in the maximum load value as compared to the control was also observed in the NC (C1), P (C4), and C (C5) groups (Figures 2A,D,E). It is assumed that the reason for the increase in the firmness of strawberries infected with selected pathogens could be the activity of bacterial consortia associated with the production of metabolites limiting mycelium growth and leading to an improvement in the quality and firmness of the fruit. Strawberries soften during ripening, mainly by degrading the middle lamina of the cell wall of the cortical parenchyma cells (Qin et al., 2017). One of the reasons for this degradation may be the activity of fungal pathogens attacking the fruit during ripening and storage (Ahmadi-Afzadi et al., 2013). The treatment of strawberries with *Hanseniaspora uvarum* yeast and infection with *B. cinerea* has been shown to preserve fruit firmness as compared to the control (Qin et al., 2017). The impact of the investigated bacterial consortia and fungal and fungal-like pathogens on the firmness of strawberries has not yet been investigated. The production of various metabolites that dissolve mycelium might play a role (Olanrewaju and Babalola, 2019). It is known that treating strawberries with beneficial microorganisms can improve the quality and properties of the stored fruit, including firmness (Fan et al., 2009). The antifungal activity of *Bacillus* sp. isolates against *C. acutatum* (Moreira et al., 2014) and *Phytophthora infestans* (Caulier et al., 2018), *Lysobacter* sp. and *Pseudomonas* sp. against *Colletotrichum* sp. (Tang et al., 2019), *S. griseus* against *P. capsici* (Nguyen et al., 2015) and *Lysobacter* sp. against *P. infestans* (Lazazzara et al., 2017) have been confirmed.

**FIGURE 3:**
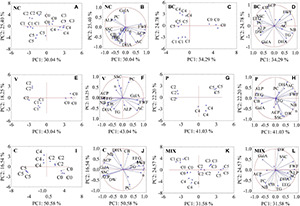
Principal component analysis (PCA) showing interrelation of microbiological consortia used and quality parameters of strawberry fruit and soil. **(A,B)** NC (no contamination), **(C,D)** BC (*B. cinerea*), **(E,F)** V (*Verticillium* sp.), **(G,H)** P (*Phytophthora* sp.), **(I,J)** C (*Colletotrichum* sp.), **(K,L)** MIX (pathogen group: BC, V, P, C.); C0, control (no bacterial consortia); C1, Consortium 1; C2, Consortium 2; C3, Consortium 3; C4, Consortium 4; C5, Consortium 5; FWF, fresh weight of fruit; DW, dry weight of fruit; SSC, soluble solid content; PC, content of phenolic compounds; AC, content of anthocyanins; GalA, content of D-galacturonic acid; n, viscosity; TG, total glomalin; EEG, easily extractable glomalin; DHA, dehydrogenase activity; ACP, acid phosphatase activity; ALP, alkaline phosphatase activity; CB, carbon inbiomass; NB, nitrogen in biomass.

Fruit firmness is closely related to the dry matter content. Their soft texture and high water content make strawberry fruit susceptible to mechanical and physical damage which facilitates the penetration of microorganisms (Siebeneichler et al., 2020). In General, fruit with a higher tissue density exhibits a higher dry matter content and a greater degree of firmness compared to fruit with a lower tissue density (Tarantino et al., 2018). This relationship was observed for the strawberries NC (C1) and C (C5), with an increase in firmness and dry matter compared to the control (Figures 2A,E, 3A,E). An inverse relationship was observed in samples P (C4) and MIX (C2), fruit with a higher degree of firmness had a lower or comparable to the control dry content (Figures 2D,F, 3D,F). It is assumed that the decrease in the firmness of fruit with a high dry matter content is caused by a relatively high cell fragility, low turgor pressure or a low degree of adhesion between the adjacent strawberry tissue cells (Harker et al., 2000). Tissue hydration has a great impact not only on firmness, but also on fresh fruit weight. Studies have shown that strawberries may contain 84–92% water, depending on the variety and cultivation method (Liu et al., 2019). Only the C3 bacterial consortium increased the fresh matter content over the no contaminated (NC) strawberry by 63% (Figure 3G) and C3 and C5 consortia increased the fresh matter content in MIX-infected strawberry by 21% on average (Figure 3L). The higher fresh weight combined with the lower dry weight values indicate a higher water content in the strawberries belonging to these groups.

One of the factors determining consumer interest in strawberries is the SSC content, which is defined as the aggregate concentration of sugars (80–90% of the SSC), acids and other solutes in the cell juice (Mackenzie et al., 2011). The average content of SSC in the tested fruits was 6% (Figures 2S–X), which is the same as other studies which reported the content of SSC in strawberries to be within the range of 4–10% (Chen H. et al., 2018). In most cases, the SSC content was greater than or comparable to the control (C0) in strawberries treated with the bacterial consortia. A statistically significant decrease in the SSC value by 0.9–3.4% compared to the control (C0) was observed in groups V (C4), C (C3), and MIX (C1 and C4) (Figures 2U,W,X). The occurrence of differences in the content of SSC in the tested strawberries is natural and may result from differences in the cultivation method (Cao et al., 2015) consisting of treating the fruit with different bacterial consortia. The SSC content is influenced by various factors, such as genetics, climate, water management and cultivation practices. Positive correlations between the nutrient content, e.g., phosphorus and an increase in SSC content was noted for the strawberries (Valentinuzzi et al., 2015). This effect may occur due to the ability of bacteria to dissolve phosphates, which lowers soil pH and increases phosphorus availability through the production of organic acids. Moreover, an increase in the content of phosphorus in the fruit was shown after the treatment of strawberry seedlings with *Bacillus* sp. and *Klebsiella planticola* (Tomic et al., 2015).

Polyphenols and anthocyanins are responsible for the bright red colour of strawberries and play a key role as bioactive ingredients (de Andrade et al., 2019). The mean content of polyphenols in the tested fruit was in the range of 93–115 mg 100 g^–1^, while the anthocyanins content was in the range of 31–57 mg 100 g^–1^. The increase in polyphenol content was only observed in the NC group (C2) and was 20% greater than in the control (C0) (Figure 2M). None of the preparations used caused a statistically significant decrease in the content of polyphenols in strawberry cv. Honeoye. Unlike the case of polyphenols, a decrease in the content of anthocyanins in strawberry NC (C2), V (C2), and MIX (C1) was noted (Figures 2M,O,R). However, an increase in anthocyanin content at the level of 25–51% compared to the control (C0) occurred only in the group of *Phytophthora* sp. infected strawberries treated with C2, C3, C4, and C5 consortia (Figure 2P). It is predicted that the changes to the level of total phenolic compounds and anthocyanins may be due to the presence of pathogenic fungi, and fungal-like-pathogens, and beneficial bacteria. The bacterial synthesis of plant hormones or amino acids may enter the shikimic acid metabolic pathway, thereby acting as the precursors of phenolic acids (Cisternas-Jamet et al., 2020). The increase in anthocyanin content may be attributed to the release of anthocyanins from degraded cellular components (López-Ortiz et al., 2019), the activity of the enzymes responsible for the synthesis of anthocyanins and also the activity of microorganisms. Lingua et al. (2013) demonstrated the positive effect of bacteria of the genus *Pseudomonas* on the anthocyanin (cyanidine-3-glucoside and pelargonidine-3-glucoside) concentration in strawberries. However, the anthocyanin content shows a high degree of variability with respect to species, varieties, growth conditions and the degree of fruit ripeness (Todeschini et al., 2018).

The content of galacturonic acid (Figure 2), which is the main component of pectin, is important in fruit processing. Pectins have stabilizing and gelling properties, which is why they are used in the production of jam, juice, drinks, and jellies (Ben Abdallah et al., 2018). In addition, galacturonic acid is extracted from fruit for the synthesis of ascorbic acid (Agius et al., 2003). The content of galacturonic acid may vary; in a previous study, a ripe strawberry of the cultivar Chandler was found to contain about 330 mg of galacturonic acid g^–1^ DW (Paniagua et al., 2017). The use of consortia resulted in an increase in the galacturonic acid content of ≥400 mg g^–1^ DW (>40%) for strawberry grown in the following variants: BC (C1), C (C2, C3, C4, and C5), and MIX (C1 and C2) (Figures 2T,W,X), which was greater than the control (C0). Relatively large fluctuations in the content of galacturonic acid confirms the complexity of the processes caused by the activity of microorganisms, which are affected by plant variety, growth conditions, pathogen infections and the consortia used.

### Rheological Properties

The rheological properties of liquids determine the behaviour of the solution at all of the production stages, such as filling, mixing, packaging and removal from the package (Lee et al., 2009). Pectin solutions are used for the production of yoghurts, jelly, juices (Ke et al., 2020), paints, pastes and medicines (Jouini et al., 2018), due to their gelling, stabilizing and flavour-enhancing properties (Ke et al., 2020).

The viscosity of 1% solutions of strawberry pectin extracted with ammonium oxalate is shown in Table 1. The increase in viscosity as compared to the control (C0) occurred in NC (C3), BC (C1, C4, and C5), C (C2), and MIX (C2, C3, C4, and C5) strawberries. Also, contamination with pathogens (BC, V, C, and MIX) significantly lowered the viscosity of the strawberry pectin solutions in the controls (C0) as compared to the control of uninfected NC strawberries (C0). These results show that the use of appropriate consortia, dedicated to a specific pathogen, can increase the viscosity of pectin solutions. The decrease in viscosity may be attributed to the activity of microorganisms that soften the fruit cell walls though pectin degradation (Mierczyńska et al., 2014). Viscosity depends, among other factors, on the molecular weight of the pectin molecules, their degree of methylation and pH, which may be affected by fungi and bacteria associated with strawberries at all stages of growth and ripening. It has been proven that viscosity increases with increasing pectin concentration (Chan et al., 2017).

**TABLE 1:** Parameters of the Ostwald–de Waele model describing the rheological properties of 1% strawberry pectin solutions.

**Contamination**	**Consortium**	**Viscosity [Pas]**	**Upward curve**			**Downward curve**		
			***k***	***n***	***R* ^2^**	***k***	***n***	***R* ^2^**
NC	C0	0.34±f0.02	0.92±d0.10	0.79±ab0.01	1.00	1.06±c0.12	0.77±ab0.01	1.00
	C1	0.20±b0.00	0.54±bcd0.23	0.80±ab0.07	1.00	0.66±a0.10	0.76±ab0.02	1.00
	C2	0.14±aa0.00	0.15±ab0.11	0.95±c0.01	1.00	0.18±b0.02	0.93±c0.01	1.00
	C3	0.42±g0.00	1.40±e0.16	0.74±a0.01	1.00	1.66±d0.21	0.74±a0.05	1.00
	C4	0.14±a0.00	0.09±a0.01	0.95±c0.02	1.00	0.11±b0.01	0.93±c0.01	1.00
	C5	0.28±d0.00	0.71±cd0.03	0.80±ab0.00	1.00	0.81±a0.09	0.78±ab0.01	1.00
BC	C0	0.10±a0.00	0.13±a0.01	0.96±b0.00	1.00	0.15±a0.01	0.94±a0.01	1.00
	C1	0.12±b0.00	0.14±a0.00	0.94±b0.00	1.00	0.16±a0.02	0.93±a0.01	1.00
	C2	–	–	–	–	–	–	–
	C3	–	–	–	–	–	–	–
	C4	0.28±c0.00	0.77±b0.07	0.78±a0.01	1.00	0.92±b0.11	0.76±c0.01	1.00
	C5	0.32±d0.00	0.79±b0.12	0.78±a0.00	1.00	1.21±c0.04	0.71±b0.02	1.00
V	C0	0.20±c0.00	0.54±a0.18	0.80±a0.05	1.00	0.67±ab0.11	0.76±a0.02	1.00
	C1	0.25±d0.00	0.63±a0.06	0.80±a0.01	1.00	0.73±b0.08	0.78±a0.01	1.00
	C2	0.15±a0.00	0.41±a0.21	0.82±a0.08	1.00	0.55±ab0.08	0.76±a0.02	1.00
	C3	0.17±b0.00	0.30±a0.02	0.85±a0.01	1.00	0.38±a0.05	0.82±a0.03	1.00
	C4	–	–	–	–	–	–	–
	C5	–	–	–	–	–	–	–
P	C0	–	–	–	–	–	–	–
	C1	–	–	–	–	–	–	–
	C2	0.03±a0.00	0.03±a0.00	0.99±a0.01	1.00	0.03±aa0.00	0.97±b0.00	1.00
	C3	–	–	–	–	–	–	–
	C4	0.28±c0.00	0.75±c0.09	0.79±b0.01	1.00	0.90±c0.12	0.76±a0.01	1.00
	C5	0.21±b0.00	0.48±b0.03	0.81±b0.00	1.00	0.55±b0.07	0.79±a0.01	1.00
C	C0	0.26±c0.00	0.78±b0.23	0.77±a0.05	1.00	0.93±c0.01	0.74±a0.01	1.00
	C1	–	–	–	–	–	–	–
	C2	0.29±d0.00	0.65±b0.05	0.81±a0.00	1.00	0.71±b0.06	0.80±b0.00	1.00
	C3	–	–	–	–	–	–	–
	C4	0.07±b0.00	0.09±a0.01	0.97±b0.01	1.00	0.11±a0.01	0.94±c0.01	1.00
	C5	0.06±a0.00	0.06±a0.00	0.10±b0.00	1.00	0.07±a0.01	0.97±d0.01	1.00
MIX	C0	0.03±b0.00	0.05±a0.01	0.96±a0.04	1.00	0.06±ab0.01	0.93±a0.01	1.00
	C1	0.02±a0.00	0.02±a0.00	0.10±a0.01	1.00	0.02±a0.00	0.98±c0.00	1.00
	C2	0.17±f0.00	0.34±b0.05	0.85±b0.02	1.00	0.40±c0.05	0.82±b0.01	1.00
	C3	0.09±d0.00	0.09±a0.00	0.97±a0.00	1.00	0.10a±b0.02	0.95±a0.01	1.00
	C4	0.06±c0.00	0.08±a0.01	0.97±a0.01	1.00	0.09±ab0.01	0.95±a0.01	1.00
	C5	0.10±e0.00	0.10±a0.00	0.96±a0.00	1.00	0.11±b0.01	0.94±a0.01	1.00

*NC, control (no contamination); BC, *B. cinerea*; V, *Verticillium* sp.; P, *Phytophthora* sp.; C, *Colletotrichum* sp.; MIX, pathogen group (BC, V, P, and C); C0, control (no bacterial consortia); C1, Consortium 1; C2, Consortium 2; C3, Consortium 3; C4, Consortium 4; C5, Consortium 5. Different letters (a–f) indicate differences between experimental variants (*P* < 0.05) as determined by the Tukey HSD test. A statistical analysis was performed for each infected group of plants separately. Data are means ± SD (*n* = 3).*

The rheological data of the 1% pectin solutions were suitably adjusted by the Ostwald–de Waele model with a high coefficient of determination of *R*^2^ > 0.996. Higher values of the coefficient k correspond to the higher viscosity of the samples. Based on the *k*-factor analysis, pectin solutions were obtained with a viscosity higher than that of the control (C0) in the NC (C3) and BC (C4 and C5) groups (Table 1). The antagonistic interactions of the microorganisms used led to the modification of pectin, an increase in apparent viscosity and the coefficient *k*, which is associated with an increase in the molar mass of pectin (Fracasso et al., 2018). The high molar mass of pectin promotes the formation of a gel structure (O’Donoghue and Somerfield, 2008). However, for some applications a lower viscosity may be seen as a positive property of the solutions due to pelargonidine lower energy consumption during processing (Venzon et al., 2015).

Flow curves reveal pseudoplastic, non-Newtonian behaviour due to the melt flow index values *n* (*n* < 1). The 1% pectin solutions tested are classified as shear thinned. Their viscosity decreases as the shear rate increases. Based on a previous study, the lower the *n* coefficient, the greater the degree of pseudoplasticity (Lee et al., 2009). The values of n for the tested solutions were in the range of 0.713–0.996. In comparison, the *n* values for 2% strawberry pectin solutions were 0.52, while for 5% strawberry pectin solutions they were 0.54 (Mierczyńska et al., 2017). Pseudoplastic fluid behaviour is important in the food industry (gelatinization of jellies and jams, thickening of juices and purees, and stabilization of liquids) (Grassino et al., 2016) and in medicine production (thickening of syrups or ointment, encapsulation of preparations) (Jouini et al., 2018).

The differences in the values of viscosity and in the flow of pectin solutions refers to the molecular properties of the samples, such as the chemical composition of neutral sugars, galacturonic acid content and the degree of pectin esterification (Fracasso et al., 2018). Microorganisms may have an indirect effect on the chemical composition of the samples (Erturk et al., 2012). The use of beneficial microorganisms such as *Pseudomonas* sp., *Bacillus* sp., and *Azospirillum* sp. results in an increase in plant biomass and fruit yields (Erturk et al., 2012). In addition, it was found that beneficial fungi and plant growth-promoting bacteria show antagonism to the pathogens *C. acutatum*, *Macrophomina phaseolina*, and *Fusarium solani* in strawberry plants (Pastrana et al., 2016). Inoculation with beneficial bacteria in order to increase plant growth also has potential environmental benefits; it could reduce the use of agricultural chemicals and facilitate adaptation to sustainable management practices (Erturk et al., 2012).

### Antagonistic Properties of Consortia Against Fungal Pathogens

In Petri dish-based assays carried out for the determination of the antifungal properties of the tested bacterial consortia, C4 and C5 did not show any clear zone of inhibition in the growth of the tested fungi, and fungal-like-pathogens or only indicated very weak inhibition (Table 2). The inhibitory effect was observed for C1, C2, and C3, with evident growth inhibition of all of the tested fungal pathogens in the case of C1 and C2, thereby confirming the antagonistic properties of the bacterial strains present in these consortia, which was observed in a greenhouse experiment. Other studies are consistent with the obtained results and confirm the antagonism of the tested bacterial consortia (contained *Bacillus* sp., *P. polymyxa*, and *Streptomyces* sp.) against strawberry pathogenic microorganisms (Chen P.H. et al., 2018; Li et al., 2021).

**TABLE 2:** Antifungal properties of the bacterial consortia used in the test expressed as the inhibition zone diameter (mm).

**Fungal pathogen/bacterial consortium**	**Inhibition zone [mm]**				
	**C1**	**C2**	**C3**	**C4**	**C5**
*Colletotrichum* sp. (C) G171/18	37.3 ± 2.5^a^	37.6 ± 2.0^a^	15.3 ± 1.5^b^	0.0 ± 0.0^d^	6.3 ± 0.6^c^
*Phytophthora* sp. (P) G408/18	56.0 ± 1.0^a^	57.6 ± 2.0^a^	48.6 ± 1.5^b^	0.0 ± 0.0^c^	0.0 ± 0.0^c^
*Verticillium* sp. (V) G296/18	40.6 ± 6.0^a^	6.6 ± 0.5^c^	15.3 ± 0.6^b^	0.0 ± 0.0^c^	14.3 ± 1.1^b^
*Botrytis cinerea* (B) G277/18	42.0 ± 6.0^a^	39.6 ± 7.6^a^	12.6 ± 2.1^b^	11.7 ± 3.0^b^	6.3 ± 0.6^b^

*Different letters (a–d) indicate differences between consortia (*P* < 0.05) as determined by the Tukey HSD test separately for each pathogen, ±standard deviation; C1, Consortium 1; C2, Consortium 2; C3, Consortium 3; C4, Consortium 4; C5, Consortium 5.*

### Influence of Microbial Activity on Soil Composition

The content of nutrients, proteins, and soil enzyme activity plays a key role in the functioning of the ecosystem. The cultivation of plants is accompanied by a decrease in soil productivity related to the depletion of nutrients, the release of toxic metabolites, and the development of pathogenic fungi (Gałązka, 2013). Total glomalin (TG) is one of the glycoproteins produced by arbuscular mycorrhizal fungi that live in symbiosis with plants and facilitate the uptake of water and nutrients from the environment. Easily extractable glomalin (EEG) has a more immunoreactive fraction than TG (Wrigth and Upadhyaya, 1998). For this reason, an increase in TG and EEG levels is desirable in the context of promoting plant growth. An increase in the TG content as compared to the control (C0) was only observed in the strawberry NC (C5), while an increase in EEG content may be observed in the strawberry BC (C5) (Table 3).

**TABLE 3:** Parameters of the quality of soil from strawberry cultivation.

**Contamination**	**Consortium**	**Total glomalin [mg g^–1^]**	**Easily extractable glomalin [mg g^–1^]**	**Dehydrogenase activity [μg TPF g^–1^ DW of soil 24 h^–1^]**	**Acid phosphatase activity [μg PNP g^–1^ DW of soil h^–1^]**	**Alkaline phosphatase activity [μg PNP g^–1^ DW of soil h^–1^]**	**Carbon in biomass [μg g^–1^]**	**Nitrogen in biomass [μg g^–1^]**
NC	C0	4.7±a0.6	2.6±a0.1	90.9±a2.1	77.1±a0.6	77.5±abc0.7	330.0±a14.4	33.0±ab1.2
	C1	5.3±ab0.3	2.9±a0.2	90.9±a2.8	74.6±a1.7	75.5±ab1.9	376.9±a8.4	34.7±ab0.4
	C2	5.0±ab0.4	3.0±a0.3	89.8±a2.6	76.7±a1.5	84.5±c2.7	308.6±a50.3	33.7±ab1.4
	C3	5.0±ab0.3	3.0±a0.1	92.5±a3.2	92.9±b0.5	69.8±a3.1	305.5±a48.5	32.4±a1.4
	C4	4.9±ab0.4	3.0±a0.0	94.0±a2.2	93.5±b2.5	71.8±ab2.6	297.8±a46.8	36.1±bc0.6
	C5	6.0±b0.5	3.0±a0.2	102.9±a3.7	88.8±c0.5	78.2±b4.3	366.1±a52.4	39.0±c1.3
BC	C0	5.6±b0.2	2.7±a0.1	90.2±c2.7	79.7±d0.3	67.2±ab5.4	384.8±a18.8	38.8±a2.3
	C1	5.4±ab0.2	2.9±ab0.2	90.5±c3.6	75.0±c1.6	78.8±c1.7	368.7±a44.7	35.0±b0.7
	C2	4.7±b0.3	2.9±ab0.3	84.7±bc1.9	85.4±a2.1	65.0±a2.7	325.0±a7.2	23.9±c0.9
	C3	5.3±ab0.3	3.1±ab0.1	80.3±ab0.9	88.9±ab0.9	80.0±c2.8	339.7±a26.2	26.2±c0.6
	C4	4.9±ab0.3	3.0±ab0.1	76.7±a3.4	93.0±b2.4	74.5±bc2.0	462.4±b3.9	32.4±b0.2
	C5	5.3±ab0.2	3.1±b0.1	78.9±ab1.0	85.1±a0.6	68.1±ab3.7	466.0±b14.6	33.2±b0.8
V	C0	4.6±a0.2	2.9±a0.0	88.7±ac2.2	83.2±a1.8	63.1±a0.9	481.5±a22.5	33.1±a1.6
	C1	5.1±a0.6	3.1±a0.1	100.3±b1.6	94.7±b2.0	63.5±a0.4	503.6±a8.9	37.8±b1.0
	C2	4.8±a0.2	3.1±a0.2	98.1±ab7.9	94.4±b3.4	61.9±a5.4	511.6±a48.3	37.7±b1.6
	C3	5.0±a0.9	3.2±a0.2	107.7±b2.1	95.4±b1.9	81.8±c2.8	477.2±a10.6	38.4±b1.2
	C4	4.0±a0.2	2.8±a0.1	96.0±ab0.9	97.2±b1.8	72.1±d1.4	446.1±a55.4	32.0±a1.5
	C5	5.3±a0.8	2.9±a0.1	82.6±c2.1	85.9±a2.0	66.6±ad2.7	448.9±a18.8	22.5±c2.6
P	C0	5.2±a0.0	2.8±a0.1	94.4±b1.5	91.7±a1.2	69.1±b1.6	483.5±ab28.0	36.8±a1.7
	C1	5.0±ab0.0	2.8±a0.1	82.3±a0.8	89.3±a3.4	65.5±b1.2	462.3±ab36.7	34.5±ab1.7
	C2	4.6±ab0.1	3.0±a0.1	86.0±a2.4	88.1±a0.5	87.3±c2.9	522.9±b15.6	31.4±b1.5
	C3	3.9±b0.3	3.0±a0.1	86.8±a4.0	95.4±a5.0	80.2±a2.3	487.0±ab30.5	32.1±b0.6
	C4	4.9±ab0.8	2.8±a0.1	85.0±a4.0	93.1±a2.6	79.2±a0.4	394.7±ac46.0	35.8±a1.1
	C5	4.7±ab0.6	2.7±a0.3	89.6±ab2.5	88.5±a3.7	78.6±a0.7	333.0±c42.5	36.0±a0.4
C	C0	4.3±a0.8	2.6±a0.1	57.7±c0.5	71.8±c2.6	59.1±a0.4	313.0±a34.7	33.7±a0.7
	C1	3.8±a0.4	2.6±a0.2	76.0±ab1.8	79.6±ac1.3	59.1±a0.3	364.7±ac13.4	38.1±b0.8
	C2	4.1±a0.4	2.6±a0.1	80.9±b1.9	80.6±a3.7	59.1±a1.1	394.5±c15.3	35.9±ab1.9
	C3	4.8±a0.2	2.5±a0.2	73.7±a1.0	86.2±ab1.3	61.6±ab1.1	455.4±d26.8	36.7±b0.2
	C4	4.4±a0.4	2.6±a0.2	101.6±d3.0	90.1±b4.6	62.9±b1.7	362.7±ac10.0	36.5±ab1.2
	C5	3.9±a0.3	2.3±a0.1	78.1±ab2.9	93.9±b3.1	70.2±c0.7	309.7±a9.6	35.9±ab0.7
MIX	C0	4.7±ab0.1	2.8±ab0.0	74.1±a3.3	92.7±b5.5	68.3±a0.9	330.0±ab2.0	36.4±a1.4
	C1	4.0±a0.3	2.7±a0.0	78.1±a2.3	84.8±ab4.7	58.5±e1.5	323.2±ab12.5	32.3±b0.9
	C2	4.4±ab0.5	2.9±ab0.1	96.5±bc4.3	86.4±ab4.2	80.8±d2.7	333.2±ab13.5	36.0±a0.4
	C3	4.4±ab0.3	3.0±ab0.1	80.4±a0.8	82.7±ac0.6	75.2±bc1.0	303.4±a13.1	33.0±b1.9
	C4	5.0±b0.3	3.1±b0.3	95.4±b1.1	88.1±ab1.6	70.1±ab1.9	347.4±b24.4	38.0±a0.4
	C5	4.6±ab0.3	2.9±ab0.2	102.7±c1.7	73.3±c1.6	79.3±cd2.9	332.0±ab17.8	31.4±b0.7

*NC, control (no contamination); BC, *B. cinerea*; V, *Verticillium* sp.; P, *Phytophthora* sp.; C, *Colletotrichum* sp.; MIX, pathogen group (BC, V, P, and C); C0, control (no bacterial consortia); C1, Consortium 1; C2, Consortium 2; C3, Consortium 3; C4, Consortium 4; C5, Consortium 5. A statistical analysis was performed for each infected group of plants separately. Data are means ± SD (*n* = 3). Different letters (a–e) indicate differences between the experimental variants (*P* < 0.05) as determined by the following statistical tests: ANOVA Tuckey (HSD) test (total glomalin: NC, BC, V, C, MIX; easily extractable glomalin: BC, V, C, MIX; dehydrogenase: BC, P, C, MIX; acid phosphatase: BC, P, C, MIX; alkaline phosphatase: NC, BC, P, MIX; carbon: NC, P, MIX; nitrogen: NC, P), F-Welch test and RIR Tuckey test for unequal numbers (Total glomalin: P; easily extractable glomalin: NC, P; dehydrogenase: V, acid phosphatase: NC, V; alkaline phosphatase: V, C; carbon: BC, V, C; nitrogen: BC, V, C, MIX), ANOVA Kruskal–Wallis test (dehydrogenase: NC).*

The data shows the significant influence of the type of infectious pathogen and the bacterial consortium used on enzyme activity. The increase in acid phosphatase content as compared to the control (C0) characterized the strawberry fruit in the groups NC (C3, C4, and C5), BC (C2, C3, and C4), V (C1, C2, C3, and C4), and C (C2, C3, C4, and C5), while an increase in the content of alkaline phosphatase occurred in groups BC (C1 and C3), V (C3 and C4), P (C2, C3, C4, and C5), C (C4 and C5), and MIX (C2, C3, and C5) (Table 3). Increased phosphatase activity is associated with a higher content of organic matter in the soil (Singh et al., 2018). In contrast to phosphatase, a statistically significant increase in dehydrogenase content was observed in strawberry V (C1 and C3), C (C1, C2, C3, C4, and C5), and MIX (C2, C4, and C5) (Table 3). The increase in dehydrogenase activity may be explained by the greater availability of nutrients (Kanchikerimath and Singh, 2001).

### Principal Component Analysis

The relationships between the parameters studied were analysed using the PCA method (Figure 3). The distribution of individual samples on the plane is shown in Figures 3A,C,E,G,I,K. The first two factors (PC1 and PC2) explain over 50% of the total variance among the analysed samples for all of the investigated variables. For all of the tested samples, the first axis (PC1) accounted for over 30% of the total variance. The samples that explain the PC1 and PC2 axis positively have the greatest impact on the tested infection factor. On this basis, we may distinguish C0 and C3 for the NC control (Figure 3A), C0 for BC (Figure 3C), C0 for V (Figure 3E), C5 for P (Figure 3G), C2 for C (Figure 3I), and C3 for MIX (Figure 3K). An analysis of the PCA1-PCA2 observation charts (Figures 3A,C,E,G,I,K) confirms the results of the analyses above; the need to select an appropriate consortium which acts antagonistically against individual pathogens in order to obtain the highest quality of fruit.

An analysis of the PCA variable charts allowed for the identification of certain dependencies between the examined parameters in all of the studied groups, a positive correlation was found between DW and SSC, which was consistent with the results of other studies (Jorquera-Fontena et al., 2014). The increase in the content of anthocyanins (AC) was accompanied by an increase in the content of phenols (PC) only in strawberries infected with *Phytophthora* sp. (Figure 3H). A positive correlation between the content of anthocyanins and polyphenols indicates a high antioxidant activity (Dembczyński et al., 2015). It was observed that an increase in total glomalin (TG) and easily extractable fractions of glomalin (EEG) in the soil were related to an increase in the level of nitrogen (NB) and carbon in biomass (CB) for the NC, V, and MIX groups (Figures 1B,F,L). Glomalin contains approximately 30% of soil carbon and is the main component of soil organic matter (Gałązka, 2013), which is associated with a positive correlation between TG and EEG and CB. The growth of glomalin-producing bacteria (TG and EEG) requires a sufficiently high nitrogen concentration thereby explaining the positive correlation between TG, EEG, and NB (Gałązka, 2013). On the other hand, it is believed that an excessively high nitrogen level in the soil leads to a significant reduction in the activity of dehydrogenases involved in the transformation of soil carbon. High dehydrogenase activity provides favourable conditions for the development of microorganisms in the soil environment (Koper and Siwik-Ziomek, 2006). A positive correlation between dehydrogenase activity (DHA) and CB was noted for the uninfected samples and those infected by *Verticillium* sp., *Colletotrichum* sp. and a mixture of pathogens (Figures 1B,F,J,L), which is consistent with the results of the studies by Turski and Wyczółkowski (2008). Our research confirms that there is only a positive correlation between acid phosphatase and alkaline phosphatase for the group infected with *Colletotrichum* sp. (Figure 3J). For the remaining groups studied, there is either a negative or an absence of any correlation between these enzymes. This situation may be explained by the development of soil microorganisms limiting the activity of certain enzymes. It is assumed that an excess of inorganic phosphorus inhibits the synthesis of phosphatases (Nahas et al., 1982).

## Conclusion

The interaction of beneficial bacteria and pathogenic fungi or fungal-like-pathogens makes it possible to preserve the high quality of strawberry fruit. The beneficial effects of the selected consortia were confirmed by comparing the results produced by infected strawberries with those of a positive control. On this basis, Consortium C2, which is composed of *Bacillus* sp. strains, was singled out as one of the most effective against selected pathogens in terms of maintaining the firmness of strawberries contaminated with a group of pathogens. There was an increase in strawberry firmness as compared to the positive control after the use of selected antifungal consortia. The microorganisms contained in the consortia also influenced an improvement in the biochemical properties of the strawberries, such as the soluble solids content and anthocyanins content. The application of consortia C2, C3, C4, and C5 was accompanied by an increase in the SSC in the fruit infected with *Verticillium* sp. as compared to the control, thereby improving the flavour of the strawberries. Consortium C5 significantly increased the level of anthocyanins in strawberries contaminated with *Phytophthora* sp. and *B. cinerea*, and in the control, it also increased the health-promoting properties of the strawberries. The impact of the tested consortia on the pectin content of the strawberries was noted. In comparing the fruit samples with untreated beneficial microorganisms (C0), it may be seen that the group of pathogens (MIX) reduced the content of D-galacturonic acid and thus pectin to the greatest extent. The following consortia: C2 (the control), C1 (*B. cinerea*), C2, C3, and C5 (*Colletotrichum* sp.) and C1 (the pathogens group) may be identified as causing the highest increase in the content of extracted pectin in infected fruit as compared to Consortium C0. The decrease in viscosity was related to the increase in pectin content, which is indicated by the negative correlation between these values for the aforementioned groups (*B. cinerea, Colletotrichum* sp., pathogens group). For consortia C2 (control), C3 and C5 (*Colletotrichum* sp.) and C1 (pathogens group) the viscosity decreased by more than one and a half times as compared to Consortium C0.

Based on our research results, we may conclude that the tested consortia show a high degree of antagonism toward the tested fungus and allow for high-quality fruit to be obtained. The use of bacterial consortia also has the potential for reducing environmental pollution and the amount of fungicide residues in the fruit. However, more research is still required to identify the most effective consortia for combating strawberry and other soft fruit fungal diseases.

## Data Availability Statement

The original contributions presented in the study are included in the article, further inquiries can be directed to the corresponding author.

## Author Contributions

MD: writing – original draft, methodology, investigation, data curation, visualization, and formal analysis. JC: writing – review and editing, conceptualization, methodology, resources, supervision, and data curation. AnG: writing – review and editing, methodology, investigation, resources, and data curation. BF-S: writing – review and editing, methodology, and resources. AM-G: methodology and investigation. LS-P: writing – review and editing, and resources. AgG: methodology, investigation, and data curation. PT: investigation. AZ: resources, supervision, and data curation. MF: writing – review and editing, conceptualization, resources, data curation, supervision, project administration, and funding acquisition. All authors contributed to the article and approved the submitted version.

## Conflict of Interest

The authors declare that the research was conducted in the absence of any commercial or financial relationships that could be construed as a potential conflict of interest.

## Publisher’s Note

All claims expressed in this article are solely those of the authors and do not necessarily represent those of their affiliated organizations, or those of the publisher, the editors and the reviewers. Any product that may be evaluated in this article, or claim that may be made by its manufacturer, is not guaranteed or endorsed by the publisher.
